# The promising role of bacteriophage therapy in managing total hip and knee arthroplasty related periprosthetic joint infection, a systematic review

**DOI:** 10.1186/s40634-023-00586-z

**Published:** 2023-02-14

**Authors:** Ahmed A. Khalifa, Sarah M. Hussien

**Affiliations:** 1 Orthopaedic Department, Qena Faculty of Medicine and University Hospital, South Valley University, Kilo 6 Qena-Safaga Highway, Qena, 83523 Egypt; 2 Qena Faculty of Medicine, South Valley University, Qena, Egypt

**Keywords:** Bacteriophage therapy, Periprosthetic infection, Total hip arthroplasty, Total knee arthroplasty

## Abstract

**Purpose:**

Total hip and knee arthroplasty periprosthetic joint infection (PJI) poses a management dilemma owing to the emergence of resistant organisms. A promising option is Bacteriophage therapy (BT) was used as an adjuvant for PJI management, aiming at treating resistant infections, decreasing morbidity, and mortality. The current review aimed to demonstrate the role and safety of using BT as an adjuvant to treat PJIs.

**Methods:**

A systematic search was performed through four databases (Embase, PubMed, Web of Science, and Scopus) up to March 2022, according to the predetermined inclusion and exclusion criteria.

**Results:**

Our systematic review included 11 case reports of 13 patients in which 14 joints (11 TKAs and three THAs) were treated. The patients’ average age was 73.7 years, underwent an average of 4.5 previous surgeries. The most common organism was the *Staphylococcus aureus* species. All patients underwent surgical debridement; for the 13 patients, eight received a cocktail, and five received monophage therapy. All patients received postoperative suppressive antibiotic therapy. After an average follow-up of 14.5 months, all patients had satisfactory outcomes. No recurrence of infection in any patient. Transaminitis complicating BT was developed in three patients, needed stoppage in only one, and the condition was reversible and non-life-threatening.

**Conclusion:**

BT is a safe and potentially effective adjuvant therapy for treating resistant and relapsing PJIs. However, further investigations are needed to clarify some BT-related issues to create effective and reproducible therapeutics. Furthermore, new ethical regulations should be implemented to facilitate its widespread use.

## Introduction

Although total hip and knee arthroplasties proved their effectiveness in improving patients’ function and life quality on long-term follow-ups, failures attributed to various modes are still occurring where periprosthetic joint infection (PJI) is considered the leading cause of total knee arthroplasty (TKA) revision, and the third common cause for revision after total hip arthroplasty (THA) [6, 28, 40]. PJI is considered one of the devastating complications after total joint arthroplasty, occurring in up to 2% of primary procedures [1]; besides the drawbacks to the patients, it poses a significant economic burden on the healthcare systems, with an even more expected increase in PJI rates owing to the expected rise of the number of primary arthroplasties which was estimated to reach 1.26 million by 2030 [54, 55].

In patients with relapsing PJI, mainly the elderly, where repeated surgeries could not be feasible, especially in the knee joint, as extensive bone loss in combination with infection might complicate the revision surgery with alternative options such as girdle stone procedures or amputation, which is usually not accepted by the patients, alternatively, a debridement, antibiotics and implant retention (DAIR procedure) followed by suppressive antibiotic therapy (SAT) could be offered in an attempt to get rid of the infection without implants removal [5, 42]; however, DAIR procedure had a variable success rate ranging from 21% to 93% [3].

The incomplete success of these surgical procedures could be attributed to the presence of a dense biofilm which hinders the conventional antimicrobial therapy from totally eradicating the bacteria [10, 22] or the emergence of antimicrobial resistance [14, 15]. A search for alternative management options led to the reconsideration of bacteriophage therapy (BT) as an adjuvant to classic antimicrobial therapy [26]. Bacteriophages are viruses that target specific bacteria and were first described in 1917; they had a good repetition in treating bone-related infections throughout the twentieth century, especially in Western Europe [2, 23, 32].

Many reports showed the potential effectiveness of BT in managing bone and joint infections [8, 23]; hence the current review aimed to document and demonstrate the role and safety of using BT as an adjuvant to treat total hip and knee arthroplasty periprosthetic joints infections.

## Methods

### Search strategy and selection criteria

A systematic search according to the Preferred Reporting Items for Systematic Reviews and Meta-Analyses (PRISMA) guidelines [38] was performed on March 2022 for articles handling the role of BT in managing PJI.

We created a search strategy based on a predefined population, intervention, comparison, and outcome (PICO) model. The population of interest was patients who had PJI (either in the hip or knee joints), the intervention was BT (or agents derived from bacteriophages such as lysin) via different administration routes, and the comparison (if present) was to standard-of-care treatments. The main outcome parameters were bacteriophage therapy’s safety and infection clearance.

A comprehensive English literature search was performed by both authors through four databases (Embase, PubMed, Web of Science, and Scopus), using various combinations of the terms “bacteriophages,” “therapy,” “periprosthetic,” and “infection.”

**Fig. 1:**
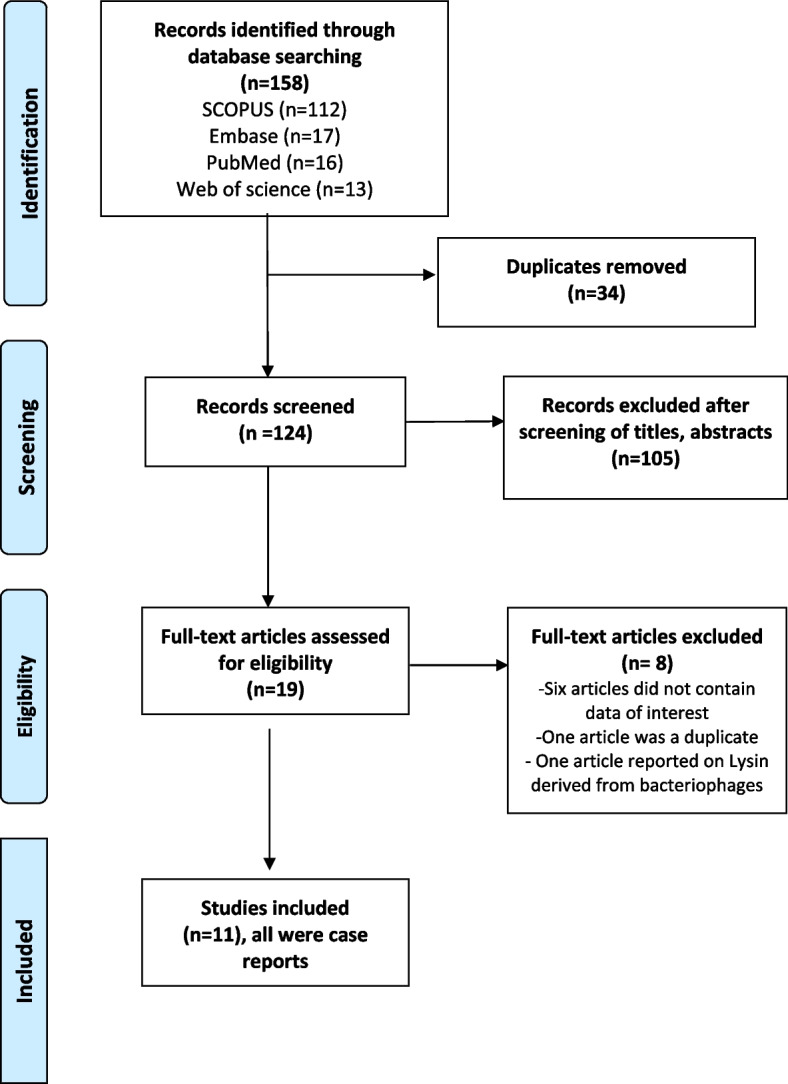
Flow diagram showing the study search and selection method

The inclusion criteria were English language studies (cohort studies, case series, and case reports) reporting on the use of bacteriophages in treating PJI in humans. Studies not published in English performed on animal models and other publication types (reviews and editorials) were excluded. After downloading the results to Endnote 20, duplicates were excluded, followed by screening the title and abstracts for eligibility. The full text of the final eligible studies was evaluated for inclusion; this resulted in 11 studies eligible for inclusion and formulation of this review (Fig. 1) [7, 11, 12, 18–21, 39, 48, 52, 56].

### Data extraction and critical appraisal

The following information was extracted from each eligible study: author(s); date of publication; country of origin; study type; the number of included patients; joint affected, comorbidities, previous surgeries; and type of organism causing the infection. For the management details, the following information was collected: type of therapy used, monophage or cocktail therapy, route of administration, period of follow up, complications, and the outcomes.

## Results

### Studies and patient characteristics (Table 1)

The 11 articles included in the current review were all case reports of 13 patients in which 14 joints (11 TKAs and three THAs) were affected (in one study, the patient had an infection in an ipsilateral THA and TKA [52]). The average age of the included patients was 73.7 (49:88) years, and the average number of previous surgeries was 4.5 (2:12).

**Table 1:** Basic characteristics of the included reports

NO.	Author	YOP	Country	Patient		CO-Morbidity	Prosthetic joint	Index surgery	No. Of previous surgeries	Organism
				Sex	Age					
1	Ferry et al.	2018	France	Female	80	obese-DM-mild chronic kidney injury	hip	NR	6	Initially phage therapy was prepared against Multidrug-resistant *Pseudomonas aeruginosa* and methicillin-susceptible *Staphylococcus aureus*. However, operative samples confirmed MSSA but not *Pseudomonas aeruginosa*, Furthermore *Enterococcus faecalis* and *Staphylococcus lugdunensis* were also detected as numerous colonies (the authors reported treating these bacteria by antibiotics).
2	Doub et al.	2020	USA	Male	72	morbid obesity-hyperlipidemia	knee	2012	6	methicillin-resistant *Staphylococcus aureus*
3	Ferry et al.	2020	France	Male	49	NR	knee	2013	2	*Staphylococcus aureus*
4	Ferry et al. (Series of 3 cases)	2020	France	1- Male	80	Parkinson disease-cardiac arrhythmia-hypertension	knee	2004	2	methicillin-susceptible *Staphylococcus aureus*
				2- Male	84	dyslipidemia	knee	2006	4	methicillin-susceptible *Staphylococcus aureus*
				3- Female	83	hypertension and lymphoedema	knee	2000	3	methicillin-susceptible *Staphylococcus aureus*
5	Tkhilaishivili et al.	2020	Germany	Female	80	DM, obesity, hypertension, and chronic kidney failure	knee	NR	4	Multi-drug resistant *Pseudomonas aeruginosa*
6	Cano et al.	2021	USA	Male	62	DM-obesity	knee	2008	12	*Klebsiella pneumoniae* complex
7	Doub et al.	2021	USA	female	79	aplastic anemia	knee	2012	4	Multi-drug resistant *Staphylococcus epidermidis*
8	Ferry et al.	2021	France	Male	88	congestive heart failure-bed ridden	knee	NR	2	relapsing *Pseudomonas aeruginosa*
9	Neuts et al.	2021	Netherlands	Male	76	NR	hip	2015	6	*Enterococcus faecalis*
10	Ramirez-Sanchez et al.	2021	USA	Female	61	NR	knee	1999	Not exactly reported (at least more than 6)	Methicillin-susceptible *Staphylococcus aureus*
11	Schoeffel et al.	2022	USA	Female	64	NR	knee	2018	4	Methicillin-resistant *Staphylococcus aureus*
							hip	2018	4	Methicillin-resistant *Staphylococcus aureus*

*YOP* Year of publication, *DM* Diabetes mellitus, *NR* Not reported

Five reports originated from the USA, four from France, one from Germany, and one from the Netherlands. In two reports, the authors mentioned that the BT was prepared or administered in another country; Neuts et al. reported that their patient had his BT in Georgia [39], while Doub et al. stated that their patient had her BT prepared in Austria [12]. For the infecting organisms, various bacterial species were defined and treated; the most common infecting organism was *Staphylococcus aureus*. Further details are shown in Table 1.

### Management details (Table 2)

In all reports, the authors mentioned that patients underwent various types of surgical procedures, including mainly debridement; however, the exact definition of these procedures differs among the included reports, especially the definition of “DAIR procedure,” which originally entails debridement with retention of the implants, and changing the modular parts (tibial insert in case of TKA or acetabular liner and femoral head in case of THA) which were performed in the report by Doub et al. [12]; however, some authors used the term “PhagoDAIR,” but they reported performing only debridement without changing the modular parts [19–21]. Furthermore, the debridement procedure was performed arthroscopically in one report [19]. In reports where the authors performed two-stage revision, the use of BT during which stage varied among reports, where it was reported to be used during the first stage only [56] and during both stages [11, 48, 52].

**Table 2:** Management details, results, and outcomes

NO.	Author		mono or cocktail BT	BT used	Route of BT administration	Follow up	BT related complications	Results and outcomes
1	Ferry et al		cocktail	PhagoDAIR: A mixture of P aeruginosa and the S aureus phages was used. Three Phages (1493, 1815, and 1957) + SAT	IA	18 months	None	The authors reported that the patient developed acute hematogenous *Citrobacter koseri* hip infection, managed by a new DAIR procedure (intraoperative samples did not grow S aureus). By the last follow up, the outcome was favorable without any clinical signs of persistent infection
2	Doub et al.		monophage	phage SaGR51Φ1 + SAT	IA (with each debridement and with final prosthesis implantation) + IV for 3 days	NR	Transaminitis related to IV phage therapy administration which was reversible and non-life-threatening. No complications of repeated IA administration.	Satisfactory outcomes, limb salvage instead of amputation, reimplantation of a Megaprostheses.
3	Ferry et al.		cocktail	phages PP1493 and PP1815	IA (Phage within DAC hydrogel)	12 months	None	Although phage therapy was used as a salvage procedure as the patient refused transfemoral amputation, The authors reported that the surgery failed due to bleeding and hematoma formation after receiving antiplatelet therapy (to treat myocardial infarction) with subsequent exposure of the free flap and re-infection; however, a bacterial culture of the hematoma revealed superinfection with *Pseudomonas aeruginosa*, Achromobacter spp., and *Proteus mirabilis* in culture. No *S. aureus* grew in culture (which was the target of the phage therapy)
4	Ferry et al. (series of 3 cases)	1	cocktail	PhagoDAIR (PP1493, PP1815, and PP1957) + SAT	IA	30 months	None	The patient had a serous discharge at 3 months which required a new DAIR procedure that showed non-specific synovitis and no infection recurrence. By the last follow up, the patient had a satisfactory outcome, limb and implant salvage, no recurrence or superinfection
		2	cocktail	PhagoDAIR	IA	7 months	None	Satisfactory outcomes, limb and implant salvage, no signs of infection, and pain-free walking.
		3	cocktail	PhagoDAIR	IA	11 months	None	Persistent discharge at 4 months required new DAIR procedures; cultures obtained during the procedure revealed no infection. By the last follow up, the patient had pain-free walking and no recurrence of infection.
5	Tkhilaishivili et al		monophage	bacteriophage therapy against MDR *P. aeruginosa* (no specific therapy was mentioned) + SAT	During the 1st stage of surgery(debridement and cement spacer), phage therapy was administered IA, followed postoperatively by IA infusion through drain tubes	10 months	None	After 2 weeks of the 1st stage of surgery, the patient experienced purulent discharge, upon which the authors decided to perform another session of debridement and change the spacer; the sample obtained during this surgery showed methicillin-resistant *Staphylococcus epidermidis* growth (treated with antibiotics) but no *P. aeruginosa* (which was the target of phage therapy). By the last follow up, the patient had satisfactory outcomes, no knee pain, functional range of motion, no loosening, and no infection recurrence.
6	Cano et al.		monophage	phage KpJH46Φ2 + SAT	IV (40 doses, each dose was taken on a weekday)	8.5 months	None	Satisfactory outcomes, limb salvage instead of amputation option, no recurrence of infection, implants were retained.
7	Doub et al		monophage	DAIR+ phage PM448 + SAT	IA + IV	5 months	A transient increase in aspartate aminotransferase (AST) and alanine aminotransferase (ALT), upon which the IV therapy was stopped owing to patient concerns and requests.	The patient was sent home after five days of the procedure. Satisfactory outcomes, limb salvage, implants were retained, and full knee function. (The authors reported that the patient was suffering from aplastic anemia, which was improved after phage therapy)
8	Ferry et al		cocktail	Arthroscopic debridement and the local application of phages, Three phages: PP1450, PP1777, and PP1792. + SAT	IA	12 months	None	By the last follow up, the knee joint was normal with painless motion and walking. No infection recurrence.
9	Neuts et al		cocktail	phages: Pyophage and IntestiPhage +SAT	oral suspension (for 19 days as a start, then a 2-week pause, followed by another 19 days of therapy).	36 months	None	Limb salvage after the patient refused the Girdlestone option, a favorable outcome, no hip complaints, no infection recurrence.
10	Ramirez-Sanchez et al		Cocktail	Three phages were used for the first treatment cycle: J-Sa36, Sa83, and Sa87. + SAT For the second treatment cycle, a single phage was used: SaGR51ø1. + SAT	For both treatment cycles, phage therapy was administered IA + IV (1st cycle was IA injection followed by IV therapy for 2 weeks) (2nd cycle was IA during the first stage of 2 stages revision)	14 months (Calculated from the 2nd treatment cycle)	None	The patient developed a recurrent infection after the 1st cycle of management, upon which she underwent two stages revision, during which she received the 2nd cycle of management. Satisfactory outcomes, limb salvage after the patient refused the above knee amputation option, no recurrence of infection, and samples from the synovial fluid showed no bacterial growth till the last follow up.
11	Schoeffel et al. knee and hip joints		monophage	Phage SaWIQ0488ø1 + SAT	IA + IV (Phage therapy was administered twice, the first was during debridement sessions for both hip and knee joints and after inserting a temporary spacer, where the patient received both IA and IV, and the second was during the implantation of the final prosthesis, during which the authors mentioned that the patient received only IA phages therapy)	11 months	A slight transaminitis did not lead to phage therapy holding and returned to normal levels after treatment stoppage.	Satisfactory outcomes, samples obtained during the final implantation of the prosthesis for both hip and knee joints revealed no bacterial growth; the patient is ambulating without a cane, able to climb stairs, and driving. No recurrence of infection.

*BT* Bacteriophage therapy, *SAT* Suppressive antibiotic therapy, *IV* Intravenous, *IA* Intraarticular, *NR* Not reported, *DAIR* Debridement, antibiotics, and implant retention, *DAC* Defensive Antibacterial Coating

Regarding the details of the BT, of the 13 patients, eight received cocktail and five received monophage therapy. The exact type of bacteriophages used for management was mentioned in ten reports (Table 2).

The route of bacteriophage therapy administration differed among reports into only intraarticular (IA) [11, 18–21, 56], only oral [39], only intravenous (IV) [7], and combined IA with IV [12, 48, 52]; furthermore, the IA administration was given by different techniques, first by direct injection in the joint cavity, second by infusion to the joint postoperatively through drainage tubes [56], and third by application with Defensive Antibacterial Coating (DAC) hydrogel on the surface of the implant [18]. All patients received postoperative suppressive antibiotic therapy (SAT) as part of their management plan for at least 6 weeks.

### Complications and outcomes (Table 2)

After an average follow up of 14.5 (5:36) months, the outcomes were satisfactory in all reports; in five reports, it was mentioned that patients refused amputation option (for TKA PJI) or girdle stone (for THA PJI) and preferred to undergo BT as a salvage procedure [7, 11, 18, 39, 48]. In all reports, no recurrence of infection was reported. However, in three reports [18, 48, 56], the authors mentioned that their patients suffered from recurrent discharge, which necessitates further debridement; in two of these reports [18, 56], the authors mentioned that the samples taken during these sessions of debridement did not reveal organisms targeted initially by the BT, in the third report, the authors did not mention details regarding the organism diagnosed during the infection recurrence [48].

Regarding bacteriophage therapy-related complications, Transaminitis was developed in three reports [11, 12, 52]; only in one report by Doub et al. the BT was stopped [11], and the condition was reversible and non-life-threatening; in the other two reports, the condition was mild and did not lead to treatment stoppage.

## Discussion

Several strategies to manage infected hip and knee total joint arthroplasties are present, mainly the DAIR procedure for early infection, single-stage revision, and two-stage revision, which showed various success rates, and possible infection recurrence [3, 31, 61]. As bacteria can develop various resistance mechanisms such as biofilm formation, which made them resistant to antimicrobial therapy [30], BT was used as an adjuvant to overcome the resistance developed against commonly used antimicrobial therapy.

Although the current review was formed only of case reports, however an increasing trend in reconsidering BT for managing resistant and recurrent total hip and knee PJI cases. Furthermore, BT showed potential efficiency in curing the infection, especially in patients with resistant or recurrent infection and in situations where revision surgery is deemed problematic, or the patients refused the other option, such as amputations. Moreover, BT showed a considerable safety profile.

Bacteriophages are non-living viruses containing DNA or RNA with a narrow activity spectrum; they differ from antibiotics as they target a specific bacterium. They could be either lytic or lysogenic; the former is the most promising for incorporation in the clinical medicine applications for infection management, as after they highjack the bacterial genome and take over the replication system, followed by further production of phages, which eventually causes bacterial lysis either through endolysin protein production or the bacterial cell wall burst [2, 11, 23]. Furthermore, following bacterial cell lysis, bacteriophages are released and start invading new bacterial cells; they continue to multiply as long as their hosting bacteria are present at a specific concentration, then the concentration will decrease gradually with bacteria elimination; this makes BT amenable to be administered as a single or few multiple doses [32]. As bacteriophages have a peculiar mode of action different from antibiotics, resistance against bacteriophages could develop at a lower incidence, unlike with various antibiotics; thus, it can treat multiple antibiotic-resistant infections [36].

The treatment challenge of PJI is related partially to biofilm-associated infections, which are usually resistant to antibiotics [57], as the biofilm forms structured communities of bacteria enabling them to survive against the host immune defense and antimicrobial therapy [10]. Bacteriophages developed innate biofilm penetration ability followed by biofilm bacterial lysis, even if the bacteria are metabolically inactive [22, 29, 60]. They also can disrupt the extracellular matrix of the biofilm using the depolymerase enzymes, making BT efficient in treating bone and joint-related reluctant infections, including biofilm formation-related infection, and becoming an attractive option for managing resistant PJIs [20, 22, 29, 41, 56, 60].

Most of the patients included in the current review underwent some surgical debridement, either open or arthroscopic; this step is helpful and synergistic for BT in many ways; first, it will help to dilute and minimize the bacterial count within the field; second, it allows for manual removal of the biofilm, third it ensures proper application of the bacteriophages near the biofilm when BT is used locally [11, 41, 56].

It is believed that the bacteria and their antagonist bacteriophages are present in nature in a balanced manner, where an increase in bacterial concentration is followed by an increase in bacteriophage concentration and vice versa [16]. Furthermore, one characteristic of bacteriophages’ action against bacteria is that it finds difficulties dealing with a low concentration of bacteria when it drops below certain levels [43, 44]; at which bacteriophages will not eradicate bacteria unless the immune system is fully functional or additive management is used; and here comes the role of suppressive antibiotic therapy (SAT) [49]. In the current review, all patients treated with BT received SAT in combination; data showed that SAT has a synergistic effect when used with BT; some believe that using BT will lower the doses and concentration of antibiotics needed, owing to a decrease in the bacterial load [2, 60]. Noteworthy that SAT should be used judiciously as if the antibiotics were given in less than optimum doses; this could lead to the emergence of bacterial variants resistant to antibiotics which subsequently make phage therapy useless [51].

Regarding the efficacy and safety of adopting BT for managing PJIs, although two of the three cases in the report by Ferry et al. developed recurrence of a discharging sinus, however, in all reports, the authors stated obtaining clearance of BT targeted infection in all patients included in the current review. Furthermore, no complications related to BT necessitating stoppage of the treatment developed in any of the patients, except for one patient who developed non-fatal transaminitis, which improved after holding the BT. Doub and Wilson further reported the occurrence of transaminitis as a complication of BT in four cases treated for resistant *S. aureus* biofilm infection [15]. In a systematic review by Clarke et al. evaluating the efficacy of BT in managing bone and joint infections, the authors reported that about 93% of the included 277 patients achieved clinical clearance of infection, with no safety concerns expressed among the included studies [8]. Furthermore, the efficacy of BT in treating infection, especially if combined with antibiotic therapy and its safety profile, was reported in the literature [2].

Although BT is an appealing option for managing resistant and relapsing PJIs, some issues and shortcomings related to bacteriophages still need to be solved; furthermore, in the reports included in the current review, we found some unclear issues which need further investigation.

First is the ethical approval for its use in light of unclear policies and regulations [17]. As in all the reports included in the current review, the authors reported that they had to obtain specific approval (expanded access) from the local authorities (such as FDA in reports from the USA and French National Agency for Medicines and Health Products Safety in reports from France), as well as approval from the institution IRB committee, and after patient gave his/her informed consent. This could be explained by the inadequate literature, documentation, and regulatory framework [46, 58]. Furthermore, there is a deficiency of well-designed clinical trials on bacteriophage use in humans, with even some conflicting evidence regarding its superiority over antibiotics or placebo [24, 33, 47]. However, in countries with no authorization for phage use as a medicine, phage therapy could be carried out under Article 37 of the Helsinki Declaration or national regulatory frameworks for treating individual patients with unauthorized treatments [37].

Second, although the idea of not developing resistant bacterial strains against BT was one of the advantages, however, the emergence of bacterial resistance is possible owing to the ability of bacteria to develop various mechanisms to prevent phage activity such as hiding, loss of receptor, producing factors which inhibit phage replication [47, 53]. This was observed with lysogenic phages, as these phages integrate into bacterial chromosomes instead of destroying them, which could possibly lead to bacteria expressing new properties related to resistance development against other phages; furthermore, when lysogenic phages integrate into bacterial cells, they can cause those bacteria to develop antibiotics resistance by acting as act as vehicles for genetic material horizontal exchange [4, 9, 47, 50]. Third, another possible challenge when determining the sensitive bacteriophages against certain bacteria is the site and method of obtaining bacterial cultures; in a pilot study by Doub et al. [14] aiming at evaluating if bacteriophage activity is the same across all in vivo PJI environments, three patients diagnosed with *S. aureus* PJI by arthrocentesis cultures and at least three deep tissue cultures, the authors tested these isolates against various BPs, they reported heterogenic bacteriophage activity depending on the type of cultures taken (arthrocentesis vs. deep tissues), the authors recommended that choosing the appropriate BT should be based on both arthrocentesis and multiple deep tissue cultures to guarantee bacteriophage activity across all in vivo environment.

Fourth, using mono or cocktail BT. As bacteriophages have a narrow spectrum of activity and high specificity, they usually act on one strain of bacteria, making them inefficient against all pathogenic strains of a single bacterial species [27, 34]. This would make the efficacy of a monophage against multi-bacterial infection questionable unless a phage cocktail contained phages active against every isolated organism. In the current review, eight patients received a cocktail and five mono BT; some authors preferred the cocktail therapy because multiple bacteriophages could expand the activity spectrum and decrease the chances of resistant development during the management course [59]. Last are the controversies related to the route of administration, dose, and duration of BT. These are attributed to lacking exact pharmacokinetic data related to BT, as they are mainly formed of proteins; there is a possibility that bacteriophages could be degraded by interacting with human metabolism, which creates the dilemma of the best administration route [34]. Furthermore, bacteriophages are known for their self-renewal capability, which was considered an advantage as phages could work like vaccines and only one dose is needed; however, this is not always the case, as some other factors (related to the patient, such as foot intake or native microbiome) could affect the self-renewal rate, making dose adjustment more challenging [35]. In the current review, Cano et al. reported that the ideal duration for IV phage therapy is unclear, as they reported normalization of CRP by the last day of therapy (which lasted for about 8 weeks), suggesting the potential need for a long management course [7]. On the contrary, Onsea et al. reported a small series of four patients who suffered from chronic osteomyelitis and were successfully treated by local cocktail phage therapy for only 7 to 10 days [41]. Furthermore, Doub et al. reported giving IV phage therapy for 3 days, which was explained by the fact that bacteriophages are capable to self-replicate, so a few days of management is only required as an adjunct to surgical debridement [11].

To overcome most of the previously reported limitations and unresolved issues related to BT, various strategies were suggested, which always starts with a call for performing well-designed controlled clinical trials aiming at validating the superiority and safety of BT; recently, the FDA approved a clinical trial in the U.S., where IV BT was used for managing drug-resistant *S. aureus*, which showed promising results [25]. Scientific meetings to discuss policies and regulations of BT usage should be held regularly [45]. Establishing bacteriophages libraries, using bacteriophages mixtures, and modifications of certain bacteriophages using genetic engineering to overcome the narrow host range [34]. For best implementation in managing PJI, Doub et al. suggested that more research should be done to identify bacteriophages with the best PJI curing capabilities, to identify the best administration route and duration of BT in cases having PJI, and more studying of the pharmacokinetic properties of bacteriophages [13].

The current review had some inherent limitations, first is the exclusive inclusion of English literature while BT is a common practice in Western Europe; this might have led to depriving the review of studies published in languages other than English. Second is the inclusion of only case reports; however, this was related to the search results based on the search terms and search engines we used. Third, we could not report on BT’s exact availability and cost as these data were lacking in the included reports.

## Conclusions

Bacteriophage therapy is an effective and safe option for treating resistant and relapsing total hip and knee arthroplasty related PJIs; it is considered a beneficial adjuvant for surgical debridement, even in cases where the implants cannot be removed. Administration of concomitant suppressive antibiotic therapy seems to be mandatory. Further investigations are needed to clarify some issues related to BT’s best route and duration; furthermore, new ethical regulations should be implemented to facilitate its widespread use.

## Data Availability

All the data related to the study are mentioned within the manuscript, however, the raw data are available with the corresponding author and will be provided upon a written request.
